# Evaluation of the “license, master, doctorate” reform in medical school of University of Lomé (Togo): strengths and weaknesses

**DOI:** 10.1186/s12909-020-02010-x

**Published:** 2020-03-31

**Authors:** Julienne Noudé TECLESSOU, Essossinam KPELAO, Bayaki SAKA

**Affiliations:** 1 Department of Dermatology, University Teaching Hospital of Lomé Togo, Lome, Togo; 2 Department of neurosurgery, University Teaching Hospital of Lomé Togo, Lome, Togo

**Keywords:** LMD - strengths, Weaknesses - medical school - Lomé (Togo)

## Abstract

**Introduction:**

The License, Master and Doctorate (LMD) reform that structured high studies in three cycles, has been instituted since the Bologna declaration in 1999. To be conformed to international standards, the LMD system has been instituted in University of Lomé in 2009 to foster pathways between medical and paramedical training. The purpose of this study was to evaluate the strengths and weaknesses of the LMD reform since its introduction in medical school of Lomé.

**Method:**

It was an opinion survey conducted during four months in University of Lomé among the medical school’s teachers about strengths and weaknesses of LMD reform since its application. The strengths were defined as all facilities brought by LMD reform in organization of courses and practices, evaluations, new Information and Communication Technologies (ICTs) (internet, video projector, courses on line). The LMD weaknesses were defined as any problem that it could generate.

**Results:**

Of 113 resident teachers of the medical school of Lomé, seventy-six have completed the questionnaire (67.2%). The majority of teachers (74) thought that the introduction of LMD reform will make Lomé medical school fit into international standards. The availability of the video projectors was mentioned by 90.8% of the teachers and 82.9% of them used it for teaching. Online course was not available. The main strengths of LMD were: a better evaluation system (33.3%), the organization of training in units with credit (28.6%), the usage of new ICTs (23.8%). Respondents also reported many weaknesses of LMD reform: the plethoric number of students (36.2%), the absence of an intermediate diploma and pathways between studies (29.3%). The Insufficiency of human resources and material was also mentioned.

**Conclusion:**

This study highlights that LMD reform needs adaptation to local realities and improvement to ensure that students will get better training in conformity with international standards.

## Introduction

The Bachelor’s degree, Master and Doctorate (Bachelor’s-Master’s-Doctorate) or LMD reform which structured high schools in three degrees was instituted since the Bologna declaration in 1999 [1]. This system is being adopted by almost all the universities in worldwide [2]. To be conformed with international standards, Africa universities joined the system distinctly [3]. In Togo, the LMD system was instituted in university of Lomé in 2009 [4]. The implementation of this reform in the medical schools is a great challenge, especially because the Bologna Declaration didn’t point out any specificity.

Previously, in medical school of Lomé, the general practitioner training was organized in three cycles without any intermediate diploma. In order to foster pathways between medical and paramedical training, the LMD reform was introduced to gather both training in bachelor’s degree and orient the students in which study according to their merit only at the end of the bachelor’s degree. Then, the LMD reorganized the medical studies into sixteen successive semesters: six for the bachelor’s degree, four for master cycle and the last six for doctorate. Courses are divided in teaching units with credits assigned. At the beginning of the third semester, practical skills in laboratory, hospital and pharmacy are also assigned with credit. After more than 10 years that it has been adopted, it seems opportune to analyze the implementation of this innovative education system. The purpose of this study was to evaluate the strengths and weaknesses of the LMD reform since its introduction in medical school of Lomé.

## Method

It was an opinion survey conducted during 4 months (from August to November 2018) in University of Lomé among the medical school’s teachers about strengths and weaknesses of LMD reform since its implementation. We included both resident professors and associates (assistant professor). Non-resident teachers were not included because they are not involved in all the activities of the faculty. A questionnaire form was addressed to them directly or by e-mail to be filled out by themselves. An explanation note about survey and the consent form were attached to the questionnaire. Participation in the survey was voluntary and anonymous. The parameters studied were general data; the strengths of the LMD reform (training on the LMD reform; organization and delivery of courses; the strengths of the LMD reform); weaknesses and appreciation of the LMD. The strengths were defined as all facilities brought by LMD reform in organization of courses and practices, evaluations, new Information and Communication Technologies (ICTs) (internet, video projector, courses on line). The LMD weaknesses were defined as any problem that the LMD system generate. Every participant gave also free opinion on how to solve weaknesses that had been identified. The data analysis was carried out using epi-info software 7.

## Results

Over 113 resident teachers of the medical school of Lomé, Seventy-six had completed the questionnaire (67.2%). The mean age was 41 years old. We observed a male predominance (92.1%) in the sample; and 43.4% were incumbent teachers. The proportion of teachers from medical and surgery department was high and estimated at 35.4 and 34.2% respectively. Those from other departments including fundamental sciences, pediatrics and gynecology, represented 19.7, 7.9, 1.3% respectively. Most of the teachers intervened in the master degree (76.3%) followed by the doctorate (56.6%) and the license (47.4%).

Most teachers (74) thought that the introduction of LMD reform will make Lomé medical school to fit into international standards. More than half (64.5%) of the teachers had not received specific training before the implementation of the LMD reform, Even though, 43 teachers (56.6%) had been trained in ICTs (Table 1) of who 30 were autodidacts. The availability of the video projectors was noted by 90.8% of the teachers and used for teaching by 82.9%. The availability of internet network in classroom was 90.8%. The handouts were issued in 89.5%. There were no online courses (Table 1). Only 15.8% teachers mentored student’s presentation after personal research on one subject. The workshop or practical works were organized by 38.2% of the teachers.

**Table 1:** LMD application in Medical school of Lomé

	Yes *n*(%)	No *n*(%)
**About LMD**		
Confirmity of international standards	74(97.4)	2 (2.6)
LMD implementation’s training	27 (35.5)	49 (64.5)
Training on ICTs	43 (56.6)	33 (43.4)
Videoprojector availability	69 (90.8)	7 (9.2)
Network availability	69 (90.8)	7 (9.2)
Online coures	0	76 (100)
Classroom availaibility	65 (85.5)	11 (14.5)
**Methods of teaching**		
Course’s Explanation	76 (100)	0
Handout Supports	68 (89.5)	8 (10.5)
Power-point Projection	63 (82.9)	13 (17.1)
Numeric Version of courses	46 (60.5)	30 (39.5)
Illustration by iconography	43 (56.6)	33 (43.4)
Student presentation	12 (15.8)	64 (84.2)
Cours dictation	2 (2.6)	74(97.4)
Practical works (workshop)	29 (38.2)	47 (61.8)

Six main strengths of LMD were cited by 63 teachers (82.9%). According to them, the system of evaluation in which the medical students were mixed with students from other faculties limited cheating (33.3%). The training organized in units with credit corresponding to the international standards was also cited. The use of new ICTs (23.8%) encouraged by LMD was additional best innovation (Table 2).

**Table 2:** the main strengths of LMD reform cited by 63 teachers

	*n*	%
Best revaluation system, little fraud	21	33.3
Study organize in teaching units with credit	18	28.6
harmonizing program among universities	18	28.6
Teaching by ICTs	15	23.8
repartition of the year in semester	12	19
Personal Research and involvement of students	7	11.1

Many weaknesses of LMD reform were noted by teachers (58) like the plethoric number of students (36.2%). Some of them (20.7%) thought that LMD reform was unsuitable and inappropriate to medical school (Table 3). The remaining problems not solved by LMD system were: the absence of intermediate diploma and pathways between cycles (29.3%), The Insufficiency of human resources (teachers, secretaries, accountants) and material (classrooms, libraries, computers, internet connection, equipped hospitals) was also mentioned (Table 3). One teacher reported that the LMD reform privileges theoretical teaching than practical.

**Table 3:** Weaknesses and pending problems of LMD noted by 58 teachers (76.3%)

	*n*	%
**Weaknesses of LMD reform**		
Increase of students number	21	36.2
Unsuitable reform	12	20.7
Insufficiency of teachers’s training	8	13.8
Absenteeism in courses	8	13.8
Absenteeism in internship	7	12.1
Decrease students level	7	12.1
Insufficiency of ICTs (network, …)	6	10.3
Difficulty of application the reform	4	6.9
Poor practical training (stage)	3	5.2
No Courses online	2	3.4
Increase of teacher’s workload	2	3.4
Multiplication of exams	2	3.4
Share classrooms with other faculties	2	3.4
**Unsolved problems**		
Absence of intermediate diploma	17	29.3
Absence of pathways between studies	17	29.3
Insufficiency of resources (human and material)	14	24.1
Poorly equipped hospitals	2	3.4

## Discussion

The main difficulty of our study was the fact that just 67.2% of the teachers filled out the questionnaire. This rate is lower than that found an Algerian, where the response rate was 100% [5]. This difference could be explained by the fact that most of the questionnaires were sent to the teachers by email. It is well known that the response rate of online surveys without financial motivations is generally between 6 and 15% lower than traditional methods (face-to-face interview) [6, 7]. In addition, it is not excluded that teachers had being reserved to judge this new LMD. The majority (64.5%) of medical school’s teachers had not received training on LMD reform since its introduction. This could limit its understanding in implementation process. The absence of training before the introduction of the LMD reform in African universities was pointed out by HUGON [8]. In Algeria [5] several university partners (teachers, students, and administrative staff) complained that the LMD reform was hasty, specifically the problem of teacher’s training. However, 56.6% of Lomé’s teachers had being trained in new ICTs. In Mali, Fomba et al. in 2011 found that only 22% of teachers had sufficient skills in computer use [9]. Methods used for teaching were handout (89.5%) and power-points (82.9%). Only 60.5% of teachers gave printed version of their courses to students. In a previous study including all the faculties of University of Lomé, the medical school was the rare faculty where new technologies were most used for teaching [4]. Our results are similar to that found by Bachir in university of Maroua [10]. To adapt, the LMD reform must use new technology. In medical school as in other faculties of University Lomé, the courses are not online [4].

According to the teachers, one of the strong points of the LMD reform in medical school was the better evaluation’s system. The evaluation in the LMD reform requires three examinations: one test in the middle of the semester, one in the end of the semester and one for the resit (the one who failed the previous evaluations). The fact that all students of the university are putting together during the evaluation can limit cheating. Most of teachers (28.6%) recognized also that the LMD reform upgrade the university of Lomé to be on international standards. This can facilitate the recognition of diplomas from University of Lomé in foreign universities [11–13]. The organization of education in the teaching units has been the core of the LMD reform in several African universities [9, 10, 14, 15]. The ICTs have revolutionized many aspects of educational system, including teaching and learning and become essential in higher education [16–18]. But they are a big challenge for most developing countries due to socio-economic and technological conditions [19].

The absence of intermediate diplomas (29.3%) and pathways between different courses of medical and paramedical training was the main weakness of LMD reform since its introduction. Indeed, in accordance with the principles of the LMD system, the first purpose of ​​the introduction of this reform in the medical schools in Togo was to combine the bachelor’s degree of all courses of health studies. The decision of admission in Master degree of any course should be made according to the average marks or scholarly achievements: the best students in research master for medical school and others in professional master for paramedical training according to merit. When this principle was first applied, all the students who validated the bachelor’s degree had refused to continue their studies in paramedical schools. This forced to reintroduce the numerus clausus in the first year to avoid the high number of medical students.

Finally, there is no intermediate diploma and pathways of students up to today as hoped. LMD reform has been successful in France with more than 6 common studies in bachelor’s degree and orientation according to the level of students [20].

The other aspects for improving LMD reform were: increase human and material resources, the availability of online courses, organization of more practical lessons and workshops, mentoring student’s presentation and homework. This situation is similar to those found in other African universities [9, 14] .

Despite these difficulties, the LMD system is not a choice for our universities, but a necessity to upgrade the training [21].

### Limitation

The main limitation of our study is related to the unwillingness of some teachers to give their opinion on the LMD reform.

## Conclusion

The introduction of LMD reform in Togo universities was done to upgrade high schools training as in worldwide. This study highlights the LMD system needs adaptation to local realities and improvement to ensure that students will get good training in concordance with international standards.

## Data Availability

The datasets used and/or analyzed during the current study available from the corresponding author on reasonable request.

## Abbreviations

LMD: License, master, and doctorate
ICTs: Information and communication technologies
